# Introduction to the virtual collection of papers on *Artificial neural networks: applications in X-ray photon science and crystallography*

**DOI:** 10.1107/S1600576723010476

**Published:** 2024-02-01

**Authors:** Tomas Ekeberg

**Affiliations:** a Department of Cell and Molecular Biology, Uppsala University, Husargatan 3 (Box 596), SE-751 24 Uppsala, Sweden

**Keywords:** artificial intelligence, AI, machine learning, deep learning, neural networks

## Abstract

Artificial intelligence is more present than ever, both in our society in general and in science. At the center of this development has been the concept of deep learning, the use of artificial neural networks that are many layers deep and can often reproduce human-like behavior much better than other machine-learning techniques. The articles in this collection are some recent examples of its application for X-ray photon science and crystallography that have been published in *Journal of Applied Crystallography*.

Artificial intelligence (AI) is more present than ever, both in our society in general and in science. At the center of this development has been the concept of deep learning, the use of artificial neural networks (ANNs) that are many layers deep and can often reproduce human-like behavior much better than other machine-learning techniques.

This development has been going on for a long time, and it is ambiguous to identify a specific starting date for it. Possible candidates might be the first ANNs in 1943 (McCulloch & Pitts, 1943 ▸), the popularization of backpropagation to train many layers in a network at once in 1985 (Rumelhart *et al.*, 1986 ▸) and the application to identify human handwriting in 1989 (Le Cun *et al.*, 1989 ▸).

Today the growth of this field is seemingly exponential, with major breakthroughs being reported almost every year. Some of the most notable examples are

(1) beating a professional player at the game of go (Silver *et al.*, 2016 ▸),

(2) residual neural networks (ResNets) for image recognition (He *et al.*, 2016 ▸),

(3) AlphaFold protein structure prediction (Jumper *et al.*, 2021 ▸),

(4) ChatGPT and other large language models (LLMs) (https://gluebenchmark.com/leaderboard).

During the same time frame, deep learning has also seeped into our society in many less prominent cases such as

(1) automatic image processing of photographs from our cell-phone cameras,

(2) recommendations for music or movies on streaming services,

(3) optimization of server hall power efficiency.

It is certainly no understatement that AI has become a foundation for society as we know it today. And so far, the technology has mainly been developed by big tech companies.

With this knowledge, many scientists have started to investigate what these new tools could mean for their field of research. While some areas of science were fairly slow to adopt these methods, we are now seeing a rapid spread of AI applications in many different fields, and the contributions in this collection of articles from *Journal of Applied Crystallography* (https://journals.iucr.org/special_issues/2024/ANNs) manifest that observation for X-ray photon science and crystallography.

Most applications of AI are, however, not of the transformative kind, comparable to the effect that AlphaFold had on the field of structure prediction. Instead, we have examples such as preprocessing that gives smaller improvements to data quality, or automation of a mundane task that would otherwise have to be done by a researcher.

*Classification.* One of the first applications of machine learning was to classify data. Famous examples include recognizing handwritten digits and letters or identifying human faces. In photon science this is also a common use case, and recently we have, for example, seen it being used for femtosecond X-ray imaging patterns (FXI) (Assalauova *et al.*, 2022 ▸), X-ray photon correlation spectroscopy (Timmermann *et al.*, 2022 ▸) and serial femtosecond crystallography (Rahmani *et al.*, 2023 ▸). This is often a convenient way to speed up a researcher’s work by automating an otherwise labor-intensive task, by classifying a small set of patterns by hand and then training a machine-learning algorithm to classify a bigger dataset in a similar fashion.

Another example is from neutron diffraction (Hao *et al.*, 2023 ▸) where a neural network approach was used not only to filter out relevant data but also to label regions of interest in each diffraction pattern.

A common critique towards neural network approaches is their black-box nature—that we usually have very limited understanding of the analysis that a trained network actually performs. A particularly interesting development is, therefore, the attempts to understand the internal workings of our networks. One such example is reported by Nawaz *et al.* (2023 ▸).

*Data analysis*. A more recent development is that neural networks take a more active role in data analysis in addition to classification. We see cases in the entire pipeline of data processing. A successful example is data preprocessing where, for example, missing regions of data can be recreated using deep neural networks. This has been shown both for small/wide-angle X-ray scattering (SAXS/WAXS) (Chavez *et al.*, 2022 ▸) and FXI (Bellisario *et al.*, 2022 ▸) experiments.

It is still fairly rare for neural networks to perform the bulk of the analysis. One such case is, however, a study of band gap structures in metal–organic frameworks where an ANN trained on a large set of simulated data was applied to experimental data with remarkable success (Gómez-Peralta *et al.*, 2022 ▸). In another example by Lim *et al.* (2023 ▸), subsurface temperatures are estimated during laser melting comparing experimental data with ANN-based simulations.

*Parameter estimation*. A more common application is the use of neural networks to provide parameters to an analysis that is otherwise using conventional methods or to reduce the search space for an existing algorithm. Chitturi *et al.* (2021 ▸) use neural networks to estimate lattice parameters for X-ray powder diffraction data, which can reduce the search space for this time-consuming algorithm by more than a factor of 100. A similar example comes from X-ray reflectivity, where a neural network was used to estimate model parameters in a fraction of the time required for traditional methods (Mareček *et al.*, 2022 ▸). Recently, a web-service called *CrystalMELA* was also released, which can estimate the crystal system using several machine-learning methods (Corriero *et al.*, 2023 ▸)

In addition to direct applications, AI is mentioned by a vast number of articles, and it is clear that there is a curiosity and optimism about AI techniques within the field. The implementation of AI techniques is, however, most likely in its infancy. Most examples included here are the work of small talented teams of researchers using relatively modest hardware. The effects are already tangible, by speeding up research, identifying hidden patterns and freeing up time for researchers by automating tasks. Given the success so far, more effort and more computational power could potentially have the same transformative effect in our field as AlphaFold has had on protein structure prediction.
